# Modified geriatric nutritional risk index in patients with pancreatic cancer: a propensity score-matched analysis

**DOI:** 10.1186/s12885-022-10071-y

**Published:** 2022-09-12

**Authors:** Teruhisa Sakamoto, Teppei Sunaguchi, Keisuke Goto, Masaki Morimoto, Yuki Murakami, Kozo Miyatani, Takehiko Hanaki, Yuji Shishido, Kyoichi Kihara, Tomoyuki Matsunaga, Manabu Yamamoto, Naruo Tokuyasu, Yoshiyuki Fujiwara

**Affiliations:** Division of Gastrointestinal and Pediatric Surgery, Department of Surgery, School of Medicine, Tottori University Faculty of Medicine, 36-1 Nishi-cho, Yonago, 683-8504 Japan

**Keywords:** mGNRI, Albumin, CRP, Pancreatic cancer

## Abstract

**Background:**

The modified nutritional geriatric risk index (mGNRI) was developed as a novel index and provides a more appropriate prognostic index than the original GNRI, which was reported to be a useful index for predicting prognoses for various malignancies. This study investigated the prognostic significance of the mGNRI compared with that of the GNRI in patients with pancreatic cancer and the association with psoas muscle volume (PMV) for survival outcomes.

**Methods:**

This retrospective study included 137 patients who had undergone pancreatectomy for pancreatic cancer. The enrolled patients were grouped as high mGNRI (≥ 85.3) or low mGNRI (< 85.3), and high GNRI (≥ 92) or low GNRI (< 92) for prognostic analysis based on cutoff values. A propensity-matched analysis was performed in this study.

**Results:**

The 5-year overall survival of patients in the high mGNRI group or high GNRI group was significantly longer than those in the low mGNRI group or low GNRI group. Statistically significant differences for the 5-year OS were observed in the three groups with respect to the combination of mGNRI and PMV. Patients with low mGNRI/low PMV had a worse 5-year OS rate compared with patients with high GNRI/high PMV or those with high GNRI or high PMV, but not both. The concordance index of the mGNRI to predict the 5-year overall survival was greater than that of the GNRI or the combination of the GNRI and PMV, but lower than that of the combination of the mGNRI and PMV. Multivariate analysis revealed that the mGNRI was an independent prognostic factor for patients with pancreatic cancer (*P* = 0.005).

**Conclusions:**

The mGNRI might be a more useful prognostic factor than the GNRI for patients with pancreatic cancer, and might predict prognostic outcomes more accurately when combined with PMV.

## Background

The nutritional status and systemic inflammatory response of patients have a striking influence on the development and progression of cancer, and a negative correlation exists between nutrition status and inflammation. Among various nutritional indicators, the geriatric nutritional risk index (GNRI) consisting of serum albumin level, and patient height and body weight was originally developed to assess the risks of mortality and morbidity in older hospitalized patients [1]. However, in the last decade, the GNRI has gained attention as a simple nutritional indicator to predict the prognosis of patients with cancer and several studies with a cohort including not only older patients but also non-older patients reported that the GNRI was a useful prognostic factor in lung cancer, esophageal cancer, gastric cancer, and pancreatic cancer [2–5]. Furthermore, we previously investigated the relationship between the GNRI and psoas muscle volume (PMV) for survival outcomes in patients with pancreatic cancer because skeletal muscle wasting was closely associated with a poor prognosis in patients with cancer. We reported that the combination of the GNRI and PMV was more useful than the GNRI alone as a prognostic factor for patients with pancreatic cancer [6].

Recently, the modified GNRI (mGNRI) incorporating C-reactive protein (CRP), a well-known acute-phase reactant in inflammatory responses, instead of serum albumin was reported to be useful for the prediction of the early recurrence and prognosis in patients with esophageal cancer [7]. Additionally, they reported that the mGNRI was a more appropriate prognostic index than the GNRI or other nutritional or inflammatory markers in esophageal cancer. However, the prognostic influence of the mGNRI in patients with pancreatic cancer remains unclear.

Therefore, instead of the GNRI, this study evaluated the significance of the mGNRI alone as a prognostic factor and the relationship between the mGNRI and PMV for prognosis prediction in patients with pancreatic cancer using propensity score-matched analysis.

## Methods

### Patients

This study included 137 patients with histologically confirmed pancreatic ductal adenocarcinoma who had undergone pancreatectomy at our hospital between July 2005 and December 2019. The resectability status of patients enrolled in this study was resectable pancreatic cancer in 135 patients and borderline resectable pancreatic cancer in 2 patients. No patient had distant metastasis in this study. The clinicopathological findings of the patients were collected from their medical records. Histopathological findings, including tumor size, lymph node involvement, and histological differentiation were classified in accordance with the 8th edition of the International Union Against Cancer Tumor-Node-Metastasis classification system [8]. All patients in this study were of Japanese ethnicity.

### Modified Geriatric Nutritional Risk Index and Geriatric Nutritional Risk Index

In accordance with a previous report, the mGNRI was calculated using the following formula: mGNRI = [14.89/CRP (mg/dL)] + [41.7 × actual body weight/ideal body weight]. The serum CRP level was set as 0.3 mg/dL when a patient’s serum CRP level was less than 0.3 mg/dL because the normal upper limit of serum CRP is 0.3 mg/dL [7].

The following formula reported by Bouillanne et al. was used to calculate the GNRI: GNRI = [14.89 × serum albumin level (g/dL)] + [41.7 × actual body weight/ideal body weight]. In accordance with their report, the cutoff value of GNRI in this study was set as 92 [1].

The values of preoperative serum CRP level, preoperative serum albumin level, and patient height and body weight obtained within 1 week prior to surgery were used in this study. The value of a patient’s actual body weight divided by the ideal body weight was set as 1 when the patient’s weight exceeded the ideal body weight [1].

### Measurement and assessment of PMV

According to our previous report, the total PMV (mm^3^) for each patient was calculated by the analysis of preoperative computed tomography images using SYNAPSE VINCENT (Fujifilm, Tokyo, Japan). Then, it was divided by the height cubed (m^3^) to produce normalized PMV values (mm^3^/m^3^). The optimal cutoff values for PMV were set to 61.5 mm^3^/m^3^ for men and 44.1 mm^3^/m^3^ for women [6].

### Statistical analysis

Chi-squared or Fisher’s exact probability tests for categorical variables, and the Mann–Whitney *U*-test for continuous variables were used to evaluate differences between two groups. A propensity score was estimated using a logistic regression model using the following covariates: age, sex, carbohydrate antigen 19–9 (CA19-9), tumor size, presence or absence of lymph node involvement, presence or absence of neoadjuvant chemotherapy, and presence or absence of adjuvant chemotherapy. Propensity score-matched analysis was performed with a caliper width of 0.25 multiplied by the standard deviation of values calculated by a logistic regression model. The 5-year overall survival (OS) curves were constructed using the Kaplan–Meier method, and differences between survival curves were examined by the log-rank test. Receiver operating characteristic analysis was used to determine the cutoff value of the mGNRI. The concordance index (C-index) was used to evaluate the mGNRI alone, the GNRI alone, and the combination of the mGNRI and PMV or GNRI and PMV to predict the 5-year OS. Univariate and multivariate analyses were performed using Cox proportional hazard regression models to determine factors with prognostic significance for OS. Variables with a *P-*value < 0.05 were included in the multivariate analysis. *P*-values less than 0.05 were considered statistically significant. All statistical analyses were performed using IBM SPSS Statistics for Windows (version 24; IBM, Armonk, NY, USA).

## Results

The median follow-up period in this study was 27.9 months (range: 1.7–177.4 months). The mean mGNRI of the patients in this study was 82.6 ± 14.2. The cutoff value of mGNRI was set at 85.3 using receiver operating characteristic curve analysis to predict the 5-year OS. Based on the cutoff value of mGNRI, the patients were divided into high mGNRI or low mGNRI groups.

Table 1 shows comparisons of the clinicopathological characteristics between the high mGNRI and low mGNRI groups before and after matching. After matching, 72 out of 137 enrolled patients remained. Significant correlations were observed between the high mGNRI group and low mGNRI group with respect to the body mass index, presence of lymph node involvement, preoperative albumin level, and preoperative lymphocyte count before matching, whereas no significant correlation was observed between the two groups with respect to the presence of lymph node involvement after matching.

**Table 1:** Comparison of clinicopathological characteristics between high and low mGNRI groups in patients with pancreatic cancer

	Before propensity score matching			After propensity score matching		
Characteristics	High mGNRI group	Low mGNRI group	*P-*value	High mGNRI group	Low mGNRI group	*P-*value
	(*n *= 91)	(*n* = 46)		(*n* = 36)	(*n* = 36)	
Age (years), median (range)	72 (47–85)	72.5 (44–85)	0.616	72.5 (55–85)	72.3 (44–85)	0.795
Sex (male), n (%)	51 (56%)	30 (65.2%)	0.302	20 (55.6%)	21 (58.3%)	0.812
Body mass index (kg/m^2^), median (range)	22.7 (18.8–32.5)	19.4 (13.7–29.4)	< 0.001	21.8 (18.9–30.0)	20.2 (13.7–28.5)	0.002
Tumor size (mm), median (range)	27.0 (11.0–85.0)	28.0 (5.0–60.0)	0.791	25.8 (11.0–85.0)	27.7 (5.0–60.0)	0.676
Tumor location (pancreatic head), n (%)	40 (44.0%)	16 (34.8%)	0.302	18 (50.0%)	14 (38.9%)	0.343
Histological grading (G2^a^ or G3^b^), n (%)	42 (46.2%)	25 (54.3%)	0.365	18 (50.0%)	20 (55.6%)	0.637
Lymph node involvement (present), n (%)	44 (48.4%)	36 (78.3%)	0.001	26 (72.2%)	26 (72.2%)	1.000
Residual tumor (present), n (%)	10 (11.0%)	3 (6.5%)	0.543	3 (8.3%)	1 (2.8%)	0.614
ASA-PS (1 or 2, %)	78 (85.7%)	33 (71.7%)	0.065	31 (86.1%)	27 (75.0%)	0.234
Preoperative albumin, g/dL, median (range)	4.1 (2.6–4.9)	3.8 (2.1–4.9)	0.001	4.1 (3.3–4.8)	3.9 (2.7–4.9)	0.039
Preoperative lymphocyte count, median (range)	1640 (140–3800)	1405 (400–6660)	0.004	1740 (140–3016)	1389 (735–2496)	0.037
Preoperative CA19-9, U/mL, median (range)	53.3 (0.7–3270.7)	106.7 (0.7–3435.0)	0.121	58.9 (1.1–1591.6)	61.0 (0.7–1446.5)	0.875
Neoadjuvant chemotherapy (present), n (%)	15 (16.5%)	4 (8.7%)	0.297	2 (5.6%)	4 (11.1%)	0.674
Adjuvant chemotherapy (present), n (%)	52 (59.1%)	28 (60.9%)	0.842	23 (63.9%)	21 (58.3%)	0.629

Continuous variables are expressed as the median and range

*mGNRI* Modified geriatric nutritional risk index, *ASA-PS* American Society of Anesthesiologists physical status, *CA19-9* Carbohydrate antigen 19–9

^a^G2: moderately-differentiated

^b^G3: poor-differentiated

**Fig. 1:**
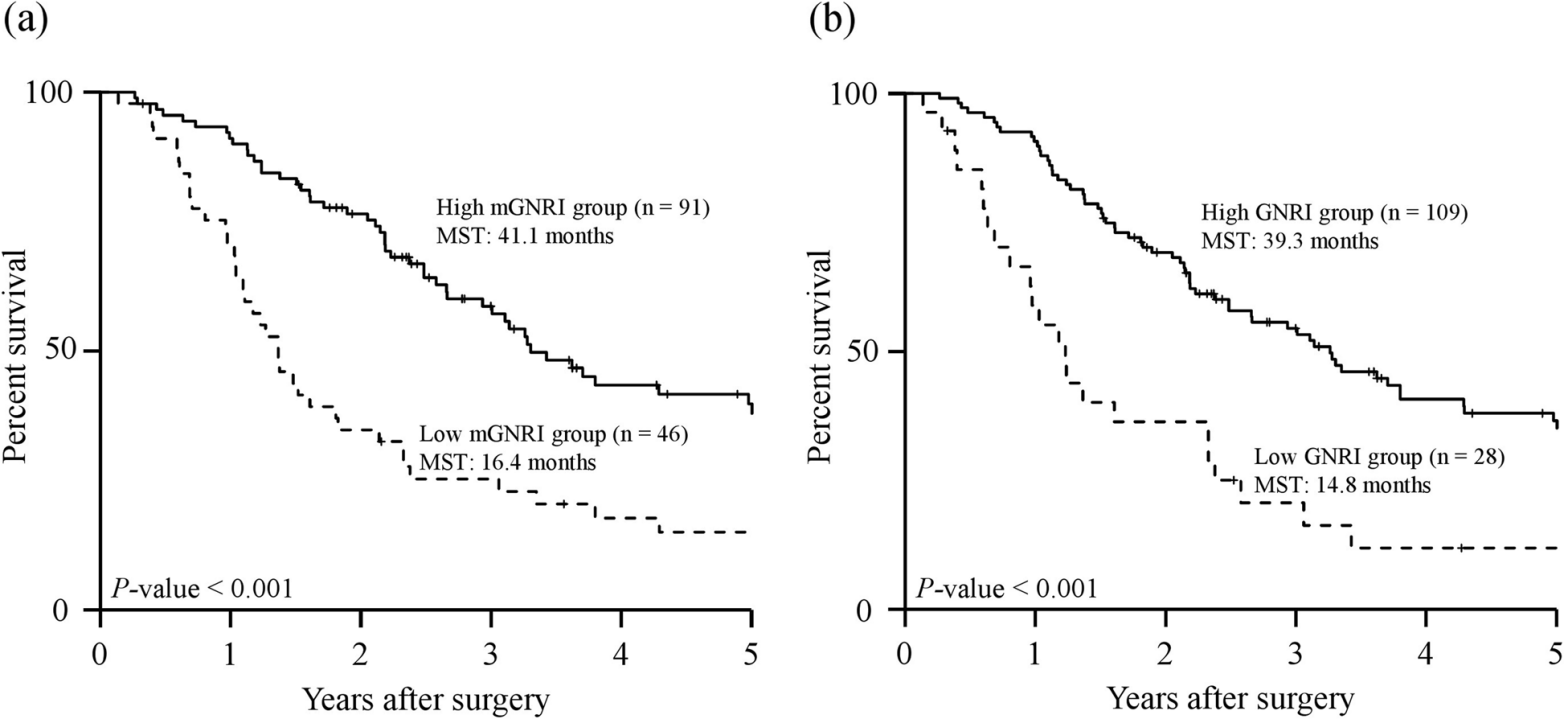
Overall survival curves in patients with pancreatic cancer based on the mGNRI (**a**) and the GNRI (**b**) before matching Abbreviations: mGNRI, modified geriatric nutritional risk index; GNRI, geriatric nutritional risk index

**Fig. 2:**
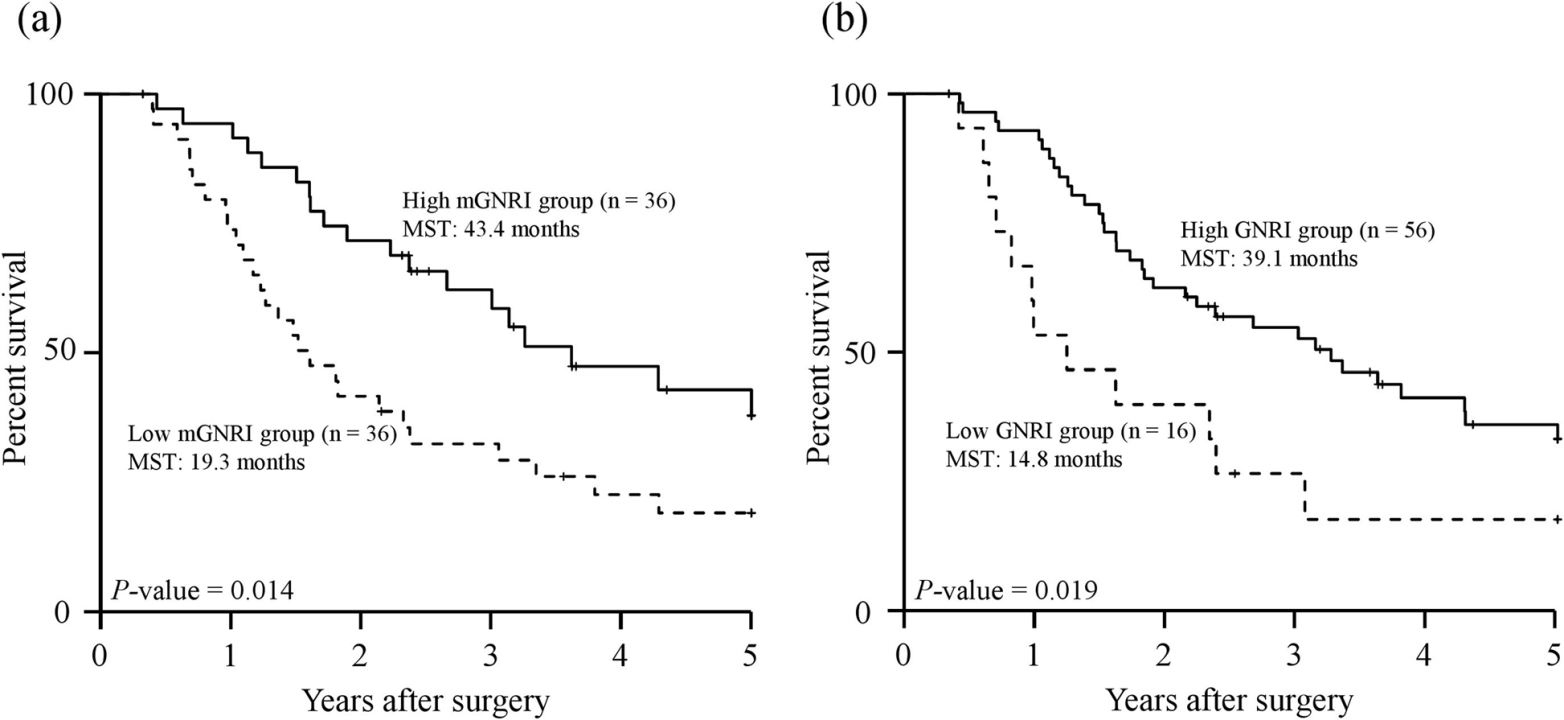
Overall survival curves in patients with pancreatic cancer based on the mGNRI (**a**) and the GNRI (**b**) after matching Abbreviations: mGNRI, modified geriatric nutritional risk index; GNRI, geriatric nutritional risk index

**Fig. 3:**
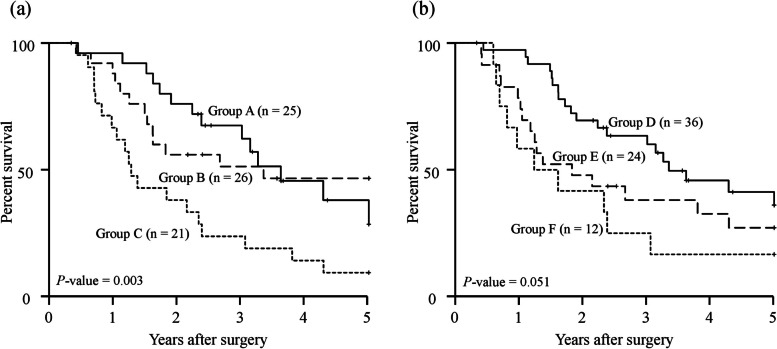
Overall survival curves in patients with pancreatic cancer compared between the combination of the mGNRI and PMV (**a**) and the combination of the GNRI and PMV (**b**) after matching. Group A, patients with high mGNRI/high PMV; group B, patients with high mGNRI or high PMV (but not both); group C, patients with low mGNRI/low PMV; Group D, patients with high GNRI/high PMV; group E, patients with high GNRI or high PMV (but not both); and group F, patients with low GNRI/low PMV. Abbreviations: mGNRI, modified geriatric nutritional risk index; PMV, psoas muscle volume; GNRI, geriatric nutritional risk index

**Fig. 4:**
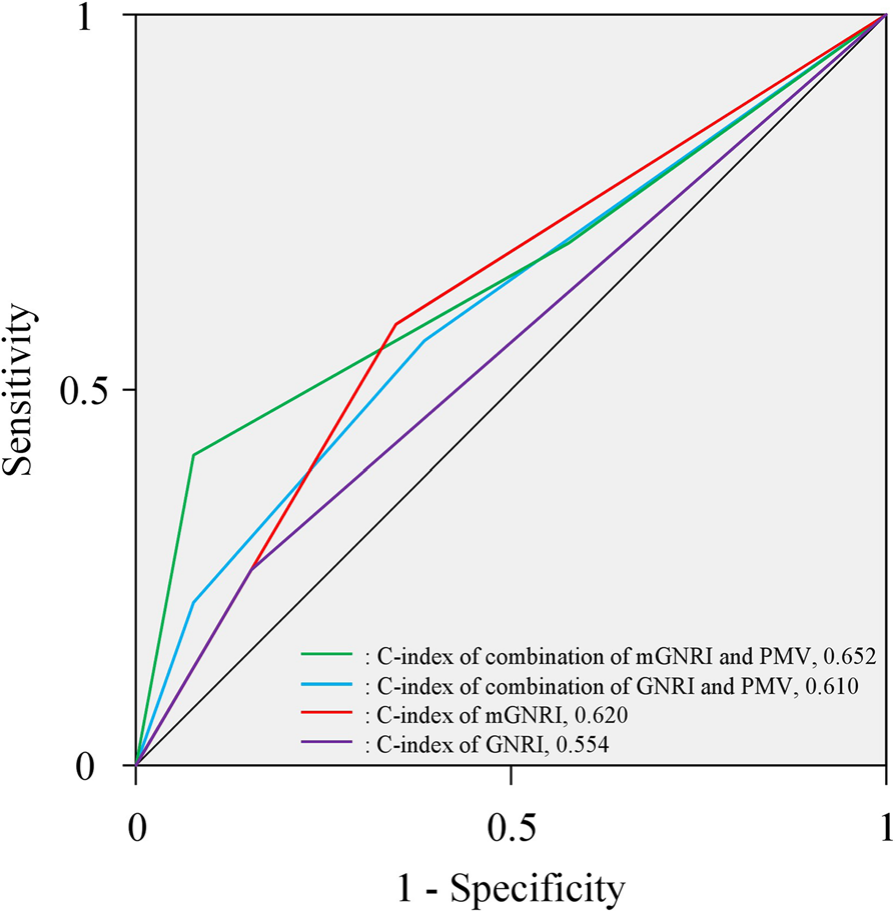
The C-index of the mGNRI, GNRI, combination of mGNRI and PMV, and combination of GNRI and PMV using the area under the curve by ROC analysis. Abbreviations: C-index, concordance index; mGNRI, modified geriatric nutritional risk index; GNRI, geriatric nutritional risk index; PMV, psoas muscle volume

The 5-year OS curves, stratified according to the mGNRI and GNRI before matching, are shown in Fig. 1a, b. The 5-year OS rate and median survival time (MST) were significantly higher in the high mGNRI group (38.7% and 41.1 months) compared with those in the low mGNRI group (16.1% and 16.4 months; *P* < 0.001, Fig. 1a). Regarding the GNRI, the 5-year OS rates in the high GNRI group were significantly higher than those in the low GNRI group (5-year OS rate: 35.9% vs 13.0%, MST: 39.3 months vs 14.8 months, respectively; *P* < 0.001, Fig. 1b). Similarly, after matching, the 5-year OS curves in the high mGNRI or high GNRI groups were significantly higher than those in the low mGNRI or low GNRI groups (mGNRI; *P* = 0.014, GNRI; *P* = 0.019, Fig. 2a, b). Furthermore, according to a combination of the mGNRI and PMV, we stratified the 72 patients into three groups after matching: A, patients with high mGNRI and high PMV (*n* = 25); B, patients with high mGNRI or high PMV (but not both) (*n* = 26); and C, patients with low mGNRI and low PMV (*n* = 21). We also stratified patients into three groups according to a combination of the GNRI and PMV: D, patients with high GNRI and high PMV (*n* = 36); E, patients with high GNRI or high PMV (but not both) (*n* = 24); and F, patients with low GNRI and low PMV (*n* = 12). Figure 3 shows the 5-year OS curves for the combination of mGNRI and PMV or the combination of GNRI and PMV. A statistically significant 5-year OS was observed for the three groups with respect to the combination of mGNRI and PMV (*P* = 0.003, Fig. 3a). However, for the combination of GNRI and PMV, the 5-year OS of patients in group D tended to be more favorable than that of patients in group E or F, although this did not reach statistical significance (*P* = 0.051, Fig. 3b). Figure 4 shows the C-index for the prediction of the 5-year OS of the mGNRI alone, GNRI alone, combination of mGNRI and PMV, and combination of GNRI and PMV using the area under the curve by ROC analysis. The C-index of mGNRI alone (0.620) was greater than that of GNRI alone (0.554) or the combination of GNRI and PMV (0.610). However, when mGNRI was combined with PMV, the C-index (0.652) was higher than that of mGNRI alone (0.620).

After matching, multivariate analysis revealed that the mGNRI was an independent prognostic factor (hazard ratio [HR]: 2.359; 95% confidence interval [CI]: 1.291–4.311; *P* = 0.005), as well as sex (HR: 2.499; CI: 1.288–4.849; *P* = 0.007), lymph node metastasis (HR: 3.953; CI: 1.723–9.066; *P* = 0.001), and American Society of Anesthesiologists physical status (ASA-PS) (HR: 2.187; CI: 1.069–4.476; *P* = 0.032) in patients with resected pancreatic cancer (Table 2).

**Table 2:** Univariate and multivariate analyses of prognostic factors for overall survival in patients with pancreatic cancer

	Univariate analysis			Multivariate analysis		
Variables	HR	95% CI	*P-*value	HR	95% CI	*P*
Age (≥ 70 vs < 70), years	0.701	0.392–1.253	0.231			
Sex (male vs. female)	2.569	1.360–4.851	0.004	2.499	1.288–4.849	0.007
BMI (< 22 kg/m^2^ vs ≥ 22 kg/m^2^)	1.384	0.754–2.541	0.295			
Tumor size (≥ 20.0 mm vs < 20.0 mm)	2.079	0.999–4.327	0.050			
Histological grading for tumor (G2^a^ or G3^b^ vs G1^c^)	1.564	0.872–2.807	0.134			
Lymph node metastasis (present vs absent)	3.650	1.619–8.228	0.002	3.953	1.723–9.066	0.001
Residual tumor (present vs absent)	2.017	0.721–5.642	0.181			
ASA-PS (3 vs 1 or 2)	2.818	1.436–5.532	0.003	2.187	1.069–4.476	0.032
Preoperative CA19-9 (≥ 274.9 U/mL vs < 274.9 U/mL)	1.425	0.687–2.957	0.342			
Neoadjuvant chemotherapy (present vs. absent)	1.164	0.416–3.260	0.773			
Adjuvant chemotherapy (present vs. absent)	0.784	0.435–1.141	0.419			
PNI (high: ≥ 47.7 vs low: < 477)	1.163	0.647–2.092	0.613			
mGNRI (high: ≥ 85.3 vs low: < 85.3)	2.060	1.143–3.713	0.016	2.359	1.291–4.311	0.005

^a^G2: moderately-differentiated

^b^G3: poor-differentiated

^c^G1: well-differentiated

*HR *Hazard ratio, *CI* Confidence interval, *BMI* Body mass index, *ASA-PS* American Society of Anesthesiologists physical status, *CA19-9* Carbohydrate antigen 19–9, *mGNRI* Modified geriatric nutritional risk index

## Discussion

This study demonstrated that mGNRI is a simple tool, which is superior to GNRI alone and the combination of GNRI and PMV for predicting the prognosis of patients with pancreatic cancer.

Pancreatic cancer patients with a low GNRI had significantly poorer prognostic outcomes than patients with high GNRI [2] [9]. Serum albumin, a protein used in clinical tests to assess nutritional condition, has been used to evaluate the morbidity and mortality of patients indicated for surgical treatment or as a prognostic predictive factor of various diseases including malignancies [10–12]. However, the American Society for Parenteral and Enteral Nutrition recently reported that serum albumin is an inflammatory marker associated with nutritional risk during nutritional assessments, and therefore, should not be used as a nutritional marker [13]. As an alternative, the GNRI is an inflammatory index, which was reported originally as a nutritional assessment tool. CRP is an acute protein involved in systemic inflammation, which can be also used as an indicator to predict survival in patients with cancer [14, 15]. A negative correlation was reported between the production of CRP and albumin, although both proteins are synthesized by hepatocytes [16, 17]. The mGNRI developed by Kouzu et al. is a novel prognostic index that uses inverse CRP instead of albumin, which is based on the reported negative correlation [7]. Furthermore, they suggested that the mGNRI was an independent prognostic factor for patients with esophageal cancer. However, the relationship between the mGNRI and prognostic outcomes in pancreatic cancer patients remains unclear. Therefore, the present study was conducted to evaluate the prognostic usefulness of the mGNRI compared with that of the GNRI in patients with pancreatic cancer. Our results found that patients with a high mGNRI had a significantly better prognosis than those with a low mGNRI and that the mGNRI was a predictive prognostic factor for patients with resected pancreatic cancer. Furthermore, the mGNRI was indicated to be more appropriate than the original GNRI when predicting the prognosis of patients with resected pancreatic cancer.

Interleukin (IL)-6 and other proinflammatory cytokines, including tumor necrosis factor, are released by inflammatory cells in the tumor microenvironment in response to tissue necrosis and the presence of tumor cells [18]. These cytokines, especially IL-6, were also shown to promote tumorigenesis by regulating multiple signaling pathways related to apoptosis, proliferation, angiogenesis, invasiveness, and metastasis [19]. Moreover, the synthesis of CRP is promoted in hepatocytes via activation by IL-6. Subsequently, CRP returns to the tumor microenvironment and promotes the autocrine growth of malignant tumors as an opsonin [18]. Therefore, serum CRP levels in patients with cancer might represent the biological behavior of cancer tissues because of the close relationship between IL-6 and CRP. In contrast, the reduced synthesis of albumin in the liver of patients with cancer is caused by the combined effects of inflammation and non-inflammatory factors such as inadequate protein and caloric intake; therefore, albumin may not reflect cancer progression as accurately as CRP in patients with cancer. Indeed, in renal cell carcinoma, CRP had a higher area under the curve for predicting OS compared with albumin [20]. These findings support the results observed in the current study.

In addition, we found that the mGNRI alone was superior to the combination of GNRI and PMV for predicting the prognosis of patients with pancreatic cancer. Body composition should also be considered to have an important role in survival outcomes in patients with cancer. We previously demonstrated that a combination of the GNRI and PMV, which are nutritional markers with distinct origins, might better predict prognosis in older patients with pancreatic cancer compared with the GNRI or PMV alone [6]. This study included older patients and non-older patients, which might have influenced these results. However, the combined mGNRI with skeletal muscle is superior to the mGNRI alone for predicting survival outcomes in patients with pancreatic cancer. These results presume that the mGNRI is more sensitive than the GNRI when reflecting prognostic outcomes in patients with pancreatic cancer.

There were several limitations in this study. It was a retrospective study with a small population of patients of East Asian ethnicity, and therefore, the findings might be biased with limited generalizability. The cutoff value of the mGNRI in this study was set at 85.3 by ROC analysis. However, the optimal cutoff value in patients with pancreatic cancer remains unclear. A large prospective study involving individuals of various ethnicities is necessary to confirm our findings.

## Conclusions

The mGNRI might be a more useful prognostic factor than the original GNRI in patients with pancreatic cancer. Furthermore, the combination of mGNRI and PMV is superior to the mGNRI alone with respect to predicting the prognosis of patients with pancreatic cancer.

## Data Availability

The datasets generated during and analyzed during the current study are not publicly available, because they contain information that could compromise the privacy of research participants, but they are available from the corresponding author on reasonable request.

## Abbreviations

GNRI: Geriatric nutritional risk index
mGNRI: Modified geriatric nutritional risk index
PMV: Psoas muscle volume
CRP: C-reactive protein
OS: Overall survival
RFS: Recurrence-free survival
C-index: Concordance index
MST: Median survival time
IL-6: Interleukin-6
